# Plasma levels of soluble ACE2are associated with sex, Metabolic Syndrome, and its biomarkers in a large cohort, pointing to a possible mechanism for increased severity in COVID-19

**DOI:** 10.1186/s13054-020-03141-9

**Published:** 2020-07-22

**Authors:** Sergey A. Kornilov, Isabelle Lucas, Kathleen Jade, Chengzhen L. Dai, Jennifer C. Lovejoy, Andrew T. Magis

**Affiliations:** Institute for Systems Biology, Seattle, WA 401 Terry Ave N, Seattle, WA 98109-5263 USA

**Keywords:** ACE2, SARS-CoV-2, COVID-19, Metabolic syndrome, GGT, Liver function

To the Editor:

Patients at high risk for mortality from COVID-19, the disease caused by severe acute respiratory syndrome coronavirus 2 (SARS-CoV-2), are more likely to be older and male and have chronic diseases such as hypertension, diabetes, cardiovascular, and chronic lung disease [1, 2]. Although the mechanisms behind these associations are poorly understood, this increased risk could be partly associated with increased expression of the cellular receptor of SARS-CoV-2, angiotensin-converting enzyme-2, found at elevated levels in older individuals, men, and in cardiovascular and inflammatory conditions [3, 4]. It maintains homeostasis of the renin-angiotensin system and converts angiotensin II to angiotensin 1-7, which has vasodilatory and anti-inflammatory properties. The membrane-bound form (mACE2) is highly expressed in the heart, airways, kidney, and liver tissue, and the enzymatically active soluble form (sACE2) is generated in response to inflammatory signals and disease via mACE2 shedding.

We interrogated the associations between plasma concentrations of sACE2 and biomarkers of metabolic syndrome (body mass index, BMI; blood pressure; glycemic markers; and lipid levels), adiposity (plasma leptin and serum adiponectin), inflammation (high-sensitivity C-reactive protein, hsCRP, white blood cell count, and interleukin-8), and liver damage (alanine aminotransferase, aspartate transaminase, and gamma-glutamyl-transferase, GGT) in a large cohort of participants in a commercial wellness program who had undergone comprehensive multi-omic profiling (*N* = 2051; 1238 women and 813 men, aged 22 to 87 years, *M* = 47.3, SD = 11.71) (see [5] for details). Clinical laboratory tests were performed in CLIA-certified laboratories by Quest Diagnostics or LabCorp. Plasma sACE2 and leptin levels were measured via proximity extension immunoassaying using Olink® Cardiovascular II proteomics panel. Analyses were performed using transformed and scaled biomarker values in a robust linear regression framework controlling for age, sex (where appropriate), 8 genetic principal components, smoking, vendor, season, use of diabetes, cholesterol-lowering, and ACE-inhibitor medications.

Confirming results from recent studies [3, 4], we found higher plasma sACE2 levels in men compared to women (*P* = 2 × 10^−16^), and in older individuals (*P* = 8.6 × 10^−11^), with the age association more pronounced in women (for the interaction, *P*_int_ = 0.02). We found higher levels of sACE2 in post-menopausal women, compared to pre-menopausal women (*P* = 0.02; see Fig. 1).

**Fig. 1:**
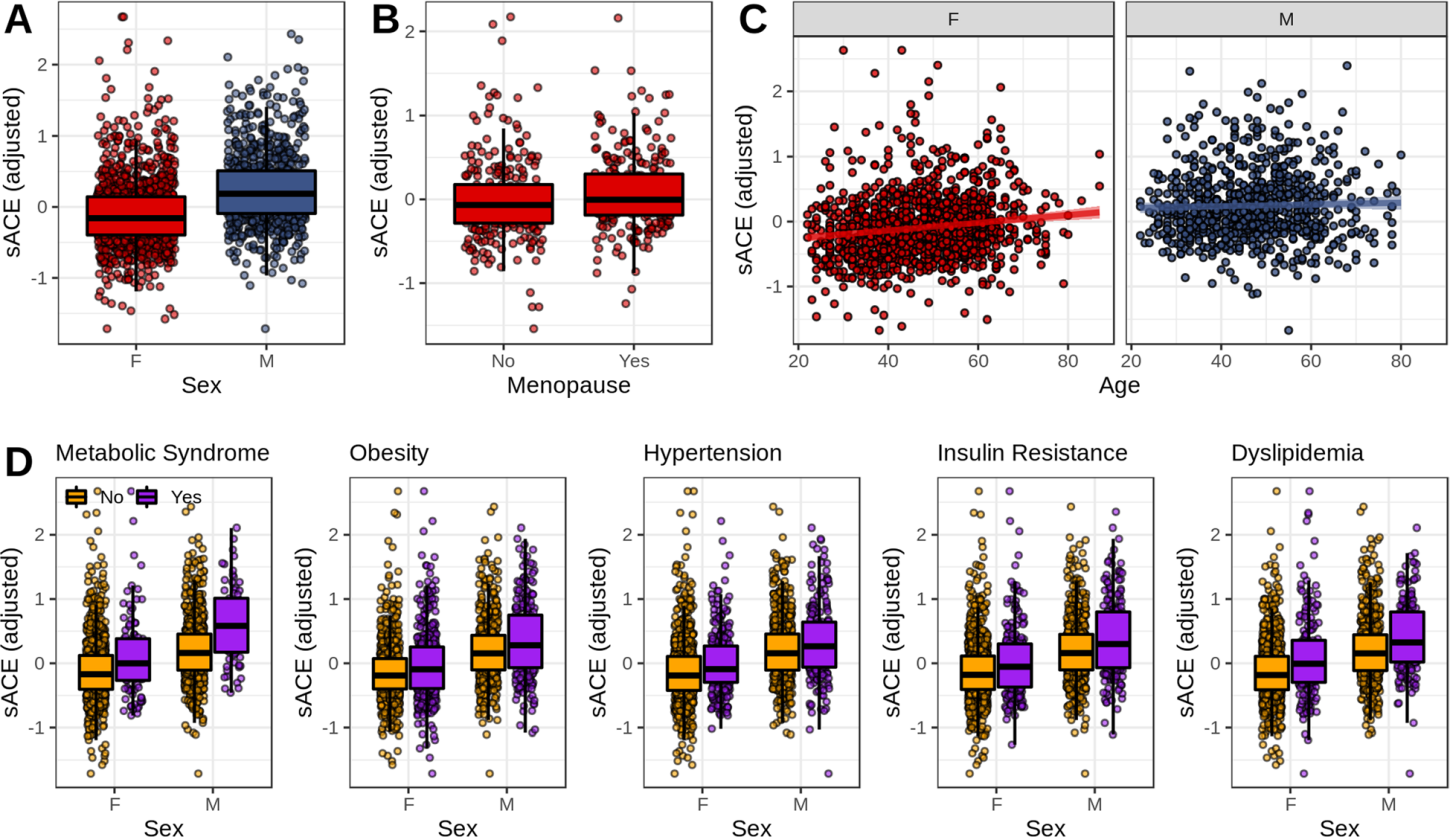
The associations of plasma sACE2 levels with sex, age, and metabolic syndrome (MetS). **a** Sex differences in plasma sACE2 levels. **b** Differences in sACE2 levels between pre- (*N* = 272) and post-menopausal (*N* = 251) women over 35 years of age. **c** Associations between sACE2 and age. **d** Differences in sACE2 levels in individuals who do vs. who do not meet diagnostic criteria for MetS and its subcomponents. The following diagnostic criteria, based on WHO guidelines, were used: obesity, BMI > 30 kg/m^2^; hyperglycemia, fasting glucose > 100 mg/dl or other evidence of insulin resistance (e.g., prescription); hypertension, systolic/diastolic blood pressure ≥ 140/90 mm/hg; dyslipidemia, triglycerides > 150 or HDL-C < 35 for men and < 39 for women. Overall status was determined as MetS if the individual met criteria for insulin resistance and satisfied at least one other domain criterion for the syndrome. sACE2, soluble ACE2 (normalized protein expression, NPX, values adjusted for covariates and scaled)

Individuals who met World Health Organization’s diagnostic criteria for metabolic syndrome (MetS) (*N* = 171) displayed elevated plasma sACE2 levels compared to controls (*N* = 1880; *P* = 4.7 × 10^− 5^); the effect was stronger in men (*P*_int_ = 8.9 × 10^− 5^). All of MetS component biomarkers were positively associated with plasma sACE2 (see Fig. 2). The associations were significantly stronger in men for biomarkers of obesity and adiposity (BMI, *P*_int_ = 0.0123; leptin, *P*_int_ = 0.0342) and insulin resistance and hyperglycemia (HbA1c, *P*_int_ = 0.0368; HOMA-IR, P_int_ = 0.042), as well as triglycerides (*P*_int_ = 0.0134) and serum hsCRP (*P*_int_ = 0.041). The strongest association was observed between sACE2 and GGT (*P* = 3.44 × 10^−90^), an important indicator of oxidative stress, liver, and bile duct damage. This association was also stronger in men (*Pint* = 0.01).

**Fig. 2:**
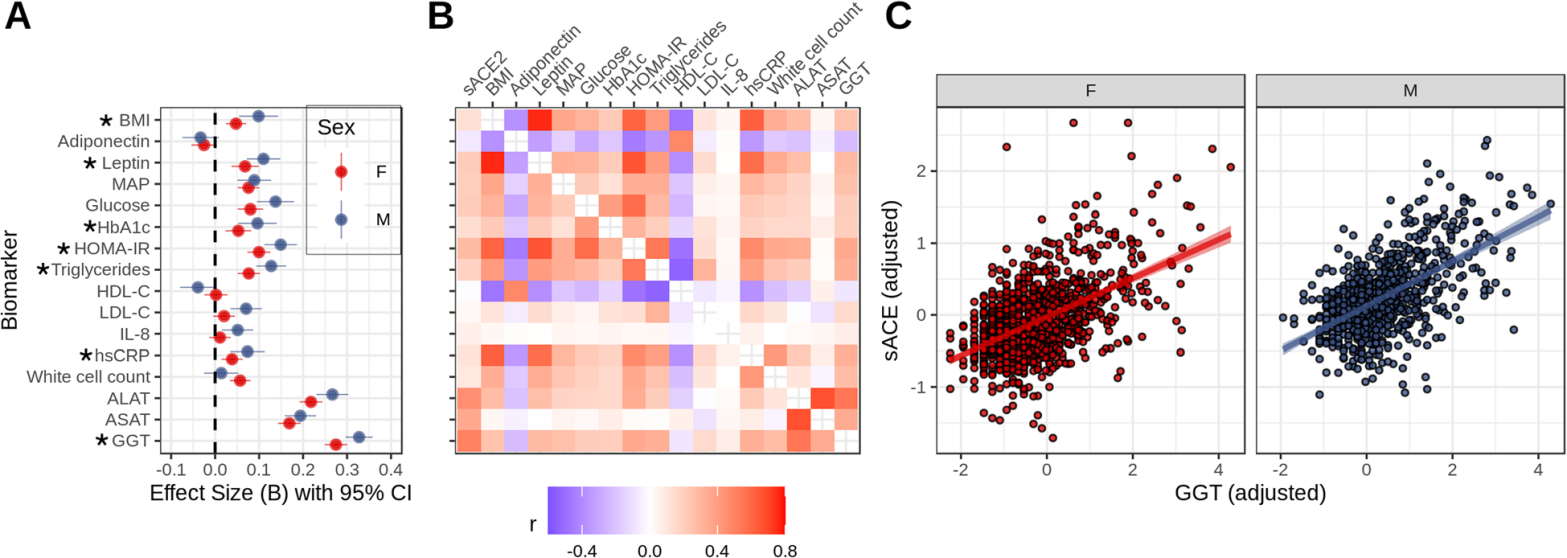
Associations between sACE2 and biomarkers of metabolic syndrome, inflammation, and liver damage. **a** Marginal Effect size (B) estimates from robust linear regressions predicting sACE2 from biomarkers estimated for men and women separately. Biomarkers for which a significant interaction with sex was established are marked with an asterisk. **b** Partial Spearman correlations between study biomarkers and sACE2 levels. **c** Scatterplot of associations between GGT and sACE2. BMI, body mass index; MAP, mean arterial blood pressure; HbA1c, glycohemoglobin A1c; HOMA-IR, homeostatic model assessment of insulin resistance; HDL-C, high-density lipoprotein cholesterol; LDL-C, low-density lipoprotein cholesterol; IL-8, interleukin 8; hsCRP, high-sensitivity C-reactive protein; ALAT, alanine aminotransferase; ASAT, aspartate transaminase; GGT, gamma-glutamyl-transferase

The robust pattern of associations between increased plasma sACE and MetS points to the possible shared pathways in cardiometabolic disease and COVID-19, implicating insulin resistance, chronic inflammation, and liver damage. This is intriguing given that both sACE2 and mACE2 have been shown to be upregulated in a rat model of chronic liver disease [6] and that sACE2 levels are higher in patients with heart failure [4]. The upregulation may be related to the tissue-specific patterns of increased SARS-CoV-2 infectivity in patients with cardiometabolic disease and/or liver damage and warrants further research on sACE2 as a potential biomarker for COVID-19 severity.

## Data Availability

The dataset supporting the conclusions of this article is available from the authors upon request.

## Abbreviations

sACE2/mACE2: Soluble/membrane-bound angiotensin-converting enzyme-2
MetS: Metabolic syndrome
GGT: Gamma-glutamyl-transferase
BMI: Body mass index
HbA1c: Glycohemoglobin A1c
HOMA-IR: Homeostatic model assessment of insulin resistance
hsCRP: High-sensitivity C-reactive protein
